# Ethical Regulation and High Intellectual Ability

**DOI:** 10.3390/ijerph19052689

**Published:** 2022-02-25

**Authors:** Sylvia Sastre-Riba, Tomás Cámara-Pastor

**Affiliations:** Department of Educational Sciences, University of La Rioja, C/Luis de Ulloa, 2, 26004 Logrono, Spain; camarapastortomas@gmail.com

**Keywords:** ethical sensitivity, giftedness, talent, regulation, moral sensitivity, executive function

## Abstract

High intellectual ability is expanding its conceptualization. This broadening includes the need for executive and ethical regulation of high potential, in order to offer effective solutions in the complexity of the 21st century. Research on the regulation of ethical sensitivity in persons with HIA is scarce and necessary, suggesting that children and adolescents with HIA are superior and earlier in ethical sensitivity than their typical peers. However, cognitive excellence does not predict excellence and its development; therefore, the importance of regulating and guiding the broad ethical sensitivity of people with HIA is highlighted. The objective of this study is to explore what is the ethical sensitivity of schoolchildren with HIA compared to typical ones. A sample of *n* = 21 schoolchildren, previously diagnosed with HIA, and an age-matched control group of *n* = 23 schoolchildren of average intelligence is studied through their answers to the ATHRI questionnaire. The multivariate general linear analysis reported intergroup differences showing the highest and earliest ethical sensitivity in schoolchildren with HIA compared to typical schoolchildren from 8 to 9 years old, but not at 10 years. The generalizability coefficient was high (0.842). Educative derivations are suggested to guide the regulation of ethical sensitivity in children.

## 1. Introduction

The complex socioeconomic, political, multicultural and technological conditions, and interdisciplinarity of knowledge in the 21st century call for complex solutions that only people with privileged minds can offer. Multidisciplinary research in high intellectual ability (HIA), recognizing the enormous personal and social capital that it entails, redefines it as a complex phenomenon multidimensionally configured by various genetic-based intellectual resources but modulated in its expression by endogenous and exogenous factors throughout the individual development. It highlights, too, the relevance of the executive and metacognitive regulation of their intellectual resources, as well as the ethical regulation of high potential [1,2] that can be used positively, or not.

In addition to the necessary advance in the knowledge of HIA, according to 21st-century research, free from the still resistant anachronisms of previous centuries such as the reliance on IQ [3], and the necessary advance in reliability diagnosis [4], or the adequacy of education to optimize the expression of potential in excellence, the emerging question is what is the role of HIA in the 21st century, and what is the value of its ethical competence to regulate high potential for the sake of human progress.

The word “regulation” takes, therefore, a necessary meaning together with the integration of a perspective that goes, beyond academic performance, towards the ethical or moral sensitivity integrated into the high logical–deductive and creative intellectual abilities. The focus today is on the 21st-century competencies that include, in addition to cognitive (see [5]), a look towards excellence [6], ethics, commitment to the common good [2,7] or wisdom [8,9], as a balance between intra, inter and contextual interests and perspectives [10].

Until now, the attention of HIA specialists has focused on building developmental models that, together with neuroscience and neuroconstructivism, identify the necessary components for the optimal expression of the potential in high intellectual productivity, creativity, knowledge or scientific advance, leaving aside the confluence of other traits necessary for this productivity to incorporate the commitment to contribute to the general well-being. Consequently, in addition to facilitating the gifted and talented individuals to develop and regulate through executive functions the high intellectual resources [1,11,12] available to make effective decisions and solve complex situations, it is necessary to incorporate ethics into it.

These regulatory abilities, beyond the cognitive ones, establish a bridge between intellectual resources and ethics with important implications in the achievement, professional decision-making and economic productivity of nations, emphasizing the importance of synthesizing the general intellectual capacity, creativity [13], wisdom [14] and ethical sensitivity to give ethical solutions in complex conditions.

### 1.1. Ethical Behavior

Ethics is closely related to Morals. It is the reflection and regulation of behavior (*ethica docens*), while Morals is the reflection and classification of behaviors (*ethica utens*) [15]. In turn, the skills for moral judgment include ethical sensitivity [16] and its development, both essential to combine them with excellence.

According to Sternberg [17], ethical behavior requires a person to: (1) recognize that they have to respond to an event or occurrence, (2) define that the event has an ethical dimension, (3) decide which is the most significant ethical dimension, (4) take responsibility to give an ethical solution to the problem, (5) discover the corresponding ethical rule, (6) decide which rule to apply, (7) resolve ethically and (8) face the possible repercussions of the action taken. Therefore, it supposes a process of self-regulation that demands a bridge between the high-level abilities with the executive functioning and the ethical values. These steps develop progressively from infancy, with wide interpersonal differences in the achievement of the process of ethical thinking and ethical behavior, so that a failure in the former entails a failure in the latter; that is, if ethical conduct is expected in the HIA, it is necessary to develop it since it is not intrinsic to HIA.

In this line, Mumford et al. [18] corroborate that ethics involves control of thought and metacognitive aspects of regulation since it involves taking into account the intentions of the other and knowing the implications of one’s own potentially influential action on others in accordance, or not, with the values of a culture. In sum, universal ethics requires a high level of cognitive maturity that supposes not only a formal procedure of resolving resources but also a preference for some options while inhibiting others, the generation of new alternatives and self-regulation, very close to the core components of executive functions.

According to Tirri [19], of the four processes related to ethical behavior, i.e., ethical sensitivity, ethical judgment, ethical motivation and ethical action, the most relevant is the first since it is essential to recognize how actions affect others, recognizing ethical problems. For this reason, it is related to moral sensitivity [20] as the ability to act, taking into account the feelings and needs of others.

Narváez and Endicott [21] have operationalized ethical sensitivity into seven skills: (1) reading and expressing emotions, (2) taking the perspective of others, (3) caring by connecting to others, (4) working with interpersonal and group differences, (5) preventing social bias, (6) generating interpretations and options and (7) identifying the consequences of action and options. To approach them metrically, they have constructed various questionnaires, including: the questionnaire Attitudes Toward Human Rights Inventory (ATHRI) [22] or the questionnaire Ethical Sensitivity Scale Questionnaire (ESSQ) [23].

### 1.2. Ethical Sensitivity and HIA

According to the foregoing, the expression and application of HIA resources require regulatory ethical skills. The relationship between them is still complex with little research [24] to clarify it, suggesting that there is greater ethical sensitivity in children, adolescents and adults with HIA [16,25] than between those of average intellectual ability. However, authors such as Ambrose [26] postulate that, despite their ethical potential, people with HIA can be influenced to develop excessively grandiose, egocentric identities and apply their potential inappropriately.

Therefore, ethical sensitivity is not an inherent characteristic of HIA [17], but it increases with greater intellectual competence since it is not possible without cognitive maturity [18]; that is, it could develop with excellence among people with HIA due to the advantage offered by its high cognitive potential. Research in this regard indicates that people with HIA have great competence in empathically capturing situations and people, high sensitivity and precision in capturing details and information that may go unnoticed by others, as well as better self-regulation of behavior with respect to typical people [27].

Although not all people with HIA have high ethical sensitivity [20], it is postulated that their cognitive development and moral reasoning is early [24] since a high percentage show greater empathy and earlier in childhood than typical children, being able to assume in an advanced way the care of others. Along the same lines, experts such as Roeper and Silverman [28] suggest the existence in them of an anticipated and more intense moral development, although they may also have potential destructive moral capacity.

Despite being necessary to continue researching the subject, the authors agree that the broad ethical sensitivity in HIA emerges from its greater cognitive awareness, broad sense of justice, empathy, insightfulness, power of observation, knowledge of consequences, moral questioning of culture and ability to imagine alternatives; in short, a combination of executive and moral management components.

Along the same lines, Urraca-Martínez et al. [29], in a comparative study of 7- to 11-year-old schoolchildren with HIA and average intelligence, corroborated the existence of greater moral sensitivity towards these issues in children with HIA compared to those of average ability, showing that the type of intellectual development (HIA vs. typical intellectual ability) and age influence the perception of world problems and their possible solutions.

All this leads to the need to establish a bridge between ethics and HIA, integrating them. In this way, high intellectual potential could be regulated and directed towards the common good by effectively leading the solutions necessary for the progress of the complex society of the 21st century. Their contribution would consist of providing tools for the resolution of social problems through ethical self-regulation of their potential, which calls for putting common needs before their own through interaction with adults who offer adequate models because, although a greater development of ethical sensitivity during childhood is found in them, there is a danger that this promise is negatively modulated by exogenous factors such as exposure to the media that do not always convey adequate values. Since academic excellence does not predict excellence and development, experts [1,19,30] stress the importance of regulating and guiding the broad ethical sensitivity of people with HIA to combine it with excellence and progress.

In accordance with the above and given the scarcity of research in the field of ethical regulation of thought in children with HIA, the objective of this study is to explore what is the ethical sensitivity of schoolchildren with HIA compared to typical schoolchildren.

## 2. Materials and Methods

### 2.1. Sample

The total sample, extracted by non-probabilistic intentional sampling, consisted of *n* = 44 participants aged 8, 9 and 10 years old. The experimental group consisted of *n* = 21 children, previously diagnosed with HIA by a professional, attending the Extracurricular Enrichment Program at the University of La Rioja, and an age-matched control group of *n* = 23 children with average intelligence attending a public school.

### 2.2. Instrument

The Attitudes Toward Human Rights Inventory (ATHRI) questionnaire [22] was designed to measure participants’ ideas about human rights and civil liberties. It was administered in the version adapted to Spanish by Cámara-Pastor [31]. Its internal validity is high (α = 0.93) according to Narváez et al. [32]. It is made up of 40 items on children’s attitudes about human rights and civil liberties grouped into 3 factors [33], F1: Personal liberties (Items 13, 18, 21, 24, 28, 36, 38) on freedoms such as birth control, freedom of expression, religion or gender; F2: Civilian constraint (Items 2, 8, 22, 30) includes attitudes towards ideological or political rights; and F3: Social security (Items 4, 6, 17, 34) includes the issue of government provision of welfare services. The response to items is using a 5-point Likert-type scale ranking from 1 (strongly agree) to 5 (strongly disagree). Higher scores on the measure indicate a greater level of ethical attitude or sensitivity.

### 2.3. Design

After extracting the study sample and having obtained the written consent of the parents of the participants, the administration of the questionnaire was carried out during school hours in groups of up to *n* = 12 schoolchildren, both in the experimental group and in the control group. The researcher with the group teacher was present during the questionnaire administration. The time taken was about 45 min.

The instruction consisted of: answering honestly to each question in the questionnaire and, in case of doubt in understanding the statement, requesting clarification from the researcher. None of the participants received any incentive. The research was carried out following the principles of the Declaration of Helsinki, with the prior written consent of the parents. The participants were fully guaranteed confidentiality.

### 2.4. Data Analysis

The data analysis consisted of: (1) calculation of descriptive statistics: mean, standard deviation and Shapiro–Wilk normality contrast test for small and independent samples, statistical significance is indicated with *p* ≤ 0.05; (2) multivariate general linear analysis (MGLM) to estimate the facets that explain the variability and the degree, taking as a fixed factor the type of intellectual development (schoolchildren with ACI vs. schoolchildren with typical intelligence) and as a response variable the results obtained in the questionnaire grouped into the factors indicated: Factor 1, Personal liberties; Factor 2, Civilian constraint; and Factor 3, Social Security, according to Crowson [33], with *p* ≤ 0.05 indicating the level of significance for rejection of the null hypothesis; (3) calculation of the generalizability of results using the G-coefficient [34], with a measurement plan in which participants constituted the instrumentation or generalization facet, while responses to the ATHRI questionnaire composed the differentiation facet.

The statistical program SPSS (v. 25.0) was used, and for the calculation of the generalizability, the program SAGT (v. 1.0) [34] was chosen.

## 3. Results

The descriptive statistical values of intergroup responses reported significantly higher mean scores in the experimental group (schoolchildren with HIA) compared to the control group, in some items, as shown in Table 1, in which Item 37 also appears with marginal significance (*p* = 0.054).

Regarding the comparison by age and intergroup (schoolchildren with HIA vs. typical schoolchildren), the results indicated statistically significant differences in the responses given at 8 years and 9 years, but not at 10 years, although the mean scores were higher in the group of children with HIA (see Table 2).

These results suggest greater ethical sensitivity at 8 years and 9 years in the group of schoolchildren with HIA compared to those in the control group, assimilating at 10 years.

Regarding the three factors in which the items are grouped [33] according to the content of ethical sensitivity, Table 3 collects the descriptive values obtained in each one of them.

As can be seen, the lowest mean was obtained in Factor F2 (*M* = 13.227), indicating that there are lower civil limits among the group with HIA, but they demand greater social responsibility and social support measures from the State. The highest response variability was that reported in F1 (Personal liberties) (*SD* = 5.427) compared to that obtained in the rest of the factors, i.e., F2 (Civilian constraint) and F3 (Social security). Perhaps the content of each of them, more or less close to their experience and understanding, could condition the response of the participants.

The normality contrast in each of the study groups and factors showed that the responses are adjusted to normality, although the distribution varies in the HIA group with respect to the control group (see Table 4).

As can be seen, in both groups the highest scores loaded in Factor F1 (Personal liberties).

**Figure 1:**
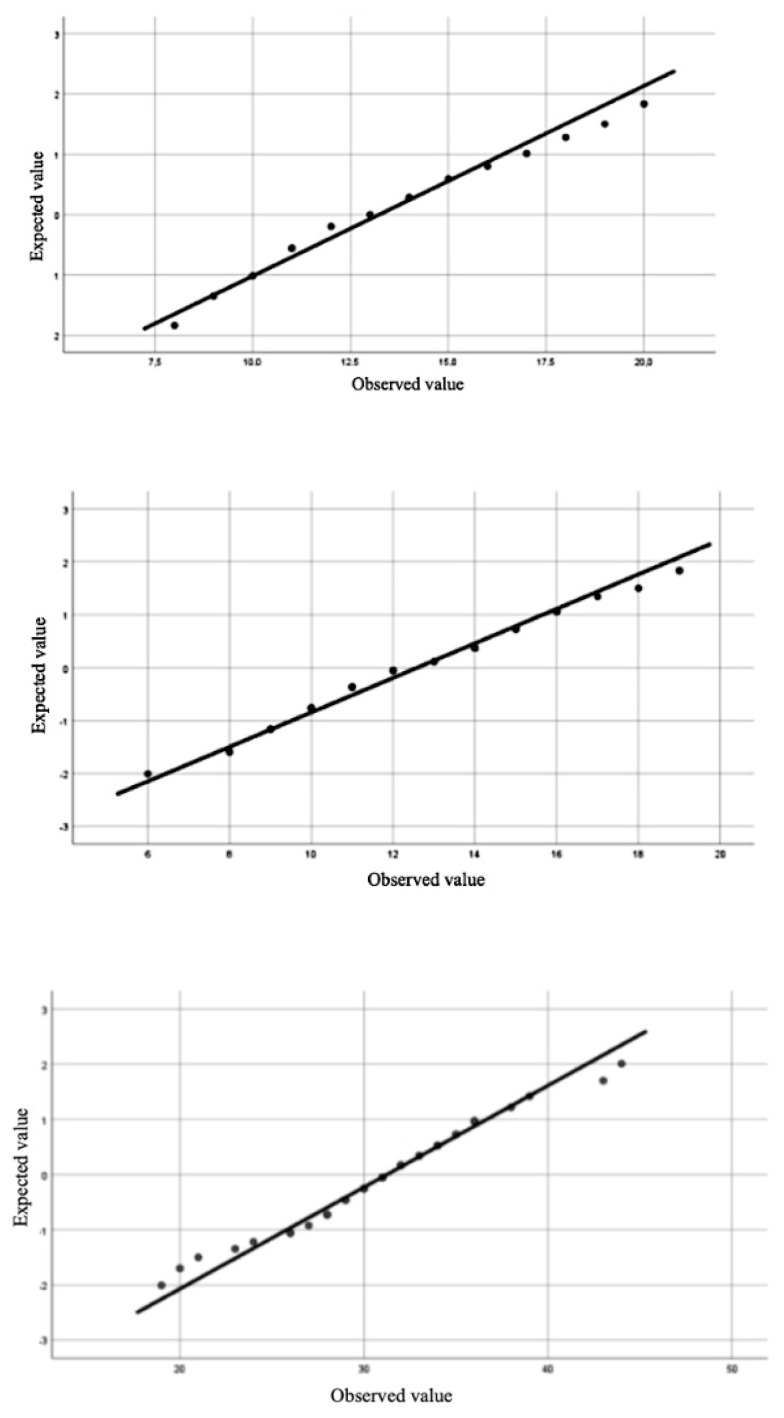
Normality distribution of the ATHRI questionnaire factors. Global sample.

For the responses of the global sample, the distribution of the scores for each of the ethical sensitivity factors F1 (*W* = 0.983, *p* = 0.734), F2 (*W* = 0.959, *p* = 0.121), F3 (*W* = 0.974, *p* = 0.420) showed that all meet the normality distribution. Figure 1 represents these results.

In conclusion, although all the factors adjusted to normality, Factor 1 is the one that obtained the best adjustment, both for the global sample (*p* = 0.734) and differentially in the group of participants with HIA (*p* = 0.830) and in the group of typical participants (*p* = 0.290).

### Intergroup Differences in Ethical Regulation

The results of the multivariate general linear analysis (MGLM) indicated the existence of statistically significant intergroup differences (schoolchildren with HIA vs. schoolchildren with typical intellectual ability) according to the Wilks lambda value (*F* = 16.087, *p* = 0.000). The effect size was high (*η2* = 0.547), indicating that the study groups are different regarding ethical sensitivity at the ages contemplated.

Table 5 collects the values obtained in the three factors studied: F1 (Personal liberties), F2 (Civilian constraint) and F3 (Social security).

As can be seen, the reported values corroborated that there are statistically significant intergroup differences in the factors F1 (*F* = 28.232, *p* = 0.000), F2 (*F* = 17,245, *p* = 0.000), and F3 (*F* = 7.952, *p* = 0.007). The effect size was large in all factors although smaller in Factor F3 (*η2* = 0.159).

The group of schoolchildren with HIA was the one that obtained statistically significant scores higher than the control group in the three factors F1 (Personal liberties) (*p* = 0.000), F2 (Civilian constraint) (*p* = 0.000), and F3 (*Social security*) (*p* = 0.007), although the effect size is smaller in this factor, as has been explained.

The generalizability coefficient was high (0.842) indicating good generalizability of the results obtained.

## 4. Discussion

The objective of this study consisted of approaching whether there were differences in the ethical sensitivity of schoolchildren with HIA and typical schoolchildren between 8 and 10 years of age. The still scarce studies on the subject [9,16,21,30,35] suggest the importance of establishing a bridge between intellectual high ability and the management of their cognitive resources applied to the effective resolution of complex problems in the society of the 21st century [24]. This calls for an ethical sensitivity [1,2] that guides the regulation in the application of these high resources.

The main results obtained in this research highlights that ethical sensitivity in HIA schoolchildren aged 8 to 10 years is higher and earlier compared to schoolchildren with typical intellectual development, according to other studies [21,28]. Concretely, results report differences in each of the factors, F1 (Personal liberties) and F2 (Civilian constraint), but Factor F3 (Social Security) did not report significant values, perhaps conditioned by the greater abstraction of the content related to the government provision of social welfare services that it comprises and that could demand greater development in ethical sensitivity to resolve them.

These results suggest that schoolchildren with HIA have greater reflection and ethical sensitivity than typical schoolchildren, probably facilitated by the complexity of their intellectual abilities, corroborating the postulates of other researchers such as Ambrose and Cross [24], Roeper and Silverman [28], Munford et al. [18], Sternberg [21], etc. They also support the conclusions obtained by Urraca et al. [29] on the perception of the world in students with HIA, showing that the perception of the world’s challenges was more complex and earlier compared to typical schoolchildren.

In sum, the children with HIA reported an earlier ability to understand and reflect on the issues that make up two of the three factors of ethical sensitivity studied: F1 (Personal liberties) and F2 (Civilian constraint); for example, the rules that govern the states, the correct actions from the incorrect ones, the implications of some world phenomena, globalization, etc.

On the other hand, the results indicated that ethical sensitivity has an earlier development among participants with HIA at 8 and 9 years but decreases at 10 years, despite the fact that mean intergroup scores remain higher in the HIA group of schoolchildren. More research is needed to explain the result that suggests this decrease, despite the earlier awareness of ethical sensitivity, in the HIA group across the ages studied. This is consistent with other authors [28,29,36], postulating that children with HIA have greater pessimism and concern for the future, as they perceive the world in a more complex and realistic way compared to their peers.

In general, however, it corroborates previous results that postulate greater development and ethical sensitivity among schoolchildren with HIA in the face of the challenges that humanity faces, suggesting a better ethical regulation of their judgment and conduct in the face of daily events. Specifically, children with HIA perceive world problems earlier and more advanced than those with typical intelligence, showing more fluidity and abstraction in it, as well as in proposing creative solutions. These results are similar to those of Ambrose [26], Ambrose and Cross, [24] or Sternberg [30], suggesting that children with HIA have a broader perception of the world, a greater sense of social justice and about the truth or lie, warning that, if they are not elaborated, they will generate responses of skepticism, loneliness and cynicism. In sum, existing studies converge on the importance of ethic sensitivity in these students and the relevance of designing educational programs that guide this sensitivity from early ages.

These results reinforce the idea of Tirri [19] that the environment and exogenous models throughout individual development, regarding ethical judgment and sensitivity, are important for schoolchildren to construct and regulate their intellectual resources ethically in favor of progress and common welfare [9]. The open question is to what extent they are prepared to understand and take advantage of the opportunities to face the challenges that emerge from the rapid and complex technological, scientific, economic and political evolution of 21st-century society [2] to achieve progress towards a better world.

In spite of its contribution, this study presents certain limitations that should be overcome in future research. For example, the small sample size due to the difficulty of its extraction, especially for participation in a study involving knowledge of the issues of sensitivity and ethical regulation of children. Another limitation has been the scarcity of formally validated instruments to know the ethical development of schoolchildren validated in Spain. It would be necessary to construct and validate a shorter and simpler instrument, best adjusted to the context, because the one used it is complicated to understand in some abstract concepts or includes sensitive or controversial issues for the age group. Another limitation might be the transversal design, which limits the understanding of changes in ethical regulation through development. Finally, it would be interesting to capture the ethical content that students receive in their usual familiar and educational contexts to estimate its impact on personal development and regulation [2,3].

These limitations encourage new lines of research and suggest the importance of transferring results. Teachers can take advantage of results obtained in order to have a better knowledge of the HIA students regarding ethical regulation as a key competence. Including this skill in education would guide the expression of high intellectual abilities combining excellence with ethics [16,19,21].

## 5. Conclusions

This work brings as the main conclusion the highest and earliest ethical sensitivity in schoolchildren with HIA compared to schoolchildren with average intelligence from 8 and 9 years old, a promise that should be adequately modulated from the educational intervention with positive ethical values and attitudes of the teachers towards them and towards its development.

Another conclusion derived is that, among the factors under study, schoolchildren with HIA show greater differences in Factors F1 (Personal liberties) and F2 (Civilian constraint), thus showing greater sensitivity to the challenges facing humanity.

Research begins to show that, although ethical sensitivity is not inherent in HIA, there is a greater possibility of developing it to greater cognitive potential and starting from the greater and earlier development of the ethical judgment available to them. For this, the study corroborates that morality and ethics are values that must be applied to the regulation of the high potential that configures the HIA in order to transfer it to progress in a complex world such as the current one. The challenge is that the greater and earlier ethical sensitivity of people with HIA crystallizes in an ethical regulation of high intellectual resources throughout development, instead of their possible decrease as the results obtained also show.

In sum, the conclusions support Renzulli’s [1,35] idea about the need of amplify the paradigm of HIA and that the nature and education of HIA in the 21st century calls for an overlap between the high cognitive resources that it entails, as well as motivational and ethical characteristics. It involves making a call for society to use resources in them so that ethical sensitivity is adequately developed, is part of their training and contributes to the regulation of high potential for its application in decision-making and resolution directed positively towards progress and the common good, as well as the regulation of one’s own behavior and towards others.

## Figures and Tables

**Table 1:** Statistically significant intergroup differences in the ATHRI questionnaire.

KERRYPNX	*F*	*p*
Item 13. Some religions should not be imposed on others that are already present in the country	4.15	0.039
Item 16. If a law was approved on equal rights between men and women, people would be disturbed for treating girls and women like boys and men	32.68	0.000
Item 17. If unemployed people can’t find work, it’s because they are not looking enough and, therefore, they should not receive financial support from the State	15.78	0.000
Item 21. Information on important issues (COVID-19, infections, birth rate, unemployment, etc.) must be available to all citizens	14.42	0.000
Item 22. Teachers must have academic freedom (teach whatever they want), even if they teach ideas not accepted by society	11.92	0.001
Item 25. We should not spend time and money on legal judgments for people of whom we are 100% sure they are guilty	4.83	0.034
Item 27. People who oppose Government policies should not be allowed to organize manifestations	2.96	0.004
Item 35. It is fair that the authorities make laws that hinder the activities of the groups protesting against a government policy or actions	19.01	0.000
Item 36. If we let atheists (people who don’t believe in any God) teach in schools, they will try to instill their ideas in the children	5.53	0.023
Item 37. The Apostolic and Roman Catholic religion must work so that women can be priest.	3.92	0.054

Note. (*p* ≤ 0.05).

**Table 2:** Significant intergroup and age differences in the ATHRI questionnaire.

KERRYPNX	*F*	*p*
8 years:		
Item 1. Politicians should encourage girls to become pilots, carpenters, military, truck drivers and other professions generally performed by men	11.667	0.011
Item 6. The Government should make it easier to get food to people living in the poorest neighborhoods of cities	5.53	0.050
Item 16. If a law was approved on equal rights between men and women, people would be disturbed for treating girls and women like boys and men	24.975	0.002
Item 21. Information on important issues (COVID-19, infections, birth rate, unemployment, etc.) must be available to all citizens	8.47	0.023
Item 40. If foreign or poor people cannot go to school because they don’t have transport, it should be provided because they have the same educational opportunities.	74.66	0.000
9 years:		
Item 2. Laws should be approved to regulate religious attitudes (how religion is lived) coming from other countries	8.62	0.008
Item 6. The Government should make it easier to get food to people living in the poorest neighborhoods of cities	15.754	0.001
Item 13. Some religions should not be imposed on others that are already present in the country	5.55	0.028
Item 27. People who oppose Government policies should not be allowed to organize manifestations	6.19	0.021
Item 34. Loyal citizens should have full basic rights, but disloyal citizens should not expect to have those rights.	15.165	0.001
10 years:		
There are not statistically significant differences	-	-

Note. (*p* ≤ 0.05).

**Table 3:** Descriptive values of the ATHRI questionnaire factors.

	*M*	*SD*
F1 (Personal liberties)	31.25	5.427
F2 (Civilian constraint)	13.227	3.176
F3 (Social security)	31.25	3.068

**Table 4:** Shapiro–Wilk normality distribution: HIA and control groups.

	HIA		Control	
	*W*	*p*	*W*	*p*
F1 (Personal liberties)	0.975	0.83	0.95	0.29
F2 (Civilian constraint)	0.965	0.611	0.934	0.136
F3 (Social security)	0.959	0.487	0.923	0.076

Note. (*p* ≤ 0.05); *W =* Shapiro–Wilk test statistic.

**Table 5:** Intergroup differences according to ethical sensitivity factors.

		*SS*	*F*	*P*	*η*2	*Post-Hoc*
Intellectual Development	F1	509.012	28.232	0.000	0.402	HIA > Control
	F2	126.249	17.245	0.000	0.291	HIA > Control
	F3	64.413	7.952	0.007	0.159	HIA > Control

Note. *SS* = sum of squares; (*p* ≤ 0.05).

## Data Availability

Data are contained within the article.

## References

[1] Renzulli J.S. (2012). Reexamining the Role of Gifted Education and Talent Development for the 21st Century. Gift. Child Q..

[2] Ambrose D., Sternberg R.J. (2016). Giftedness and Talent in the 21st Century: Adapting to the Turbulence of Globalization.

[3] Sternberg R.J. (2020). Transformational Giftedness: Rethinking Our Paradigm for Gifted Education. Roeper Rev..

[4] Riba S.S., Tarrida A.C. (2017). Fiabilidad y estabilidad en el diagnóstico de la alta capacidad intelectual. Rev. Neurol..

[5] Bellanca J., Brandt R. (2010). 21st Century Skills: Rethinking How Students Learn.

[6] Sastre-Riba S. (2013). High Intellectual Ability: Extracurricular enrichment and cognitive manageme. J. Educ. Gift..

[7] Seider S., Davis K., Gardner H., Ambrose D., Cross T. (2009). Morality, Ethics and Good Work: Young People’s Respectful and Ethical Minds. Morality, Ethics and Gifted Minds.

[8] Sternberg R.J. (1998). A Balance Theory of Wisdom. Rev. Gen. Psychol..

[9] Sternberg R.J., Sternberg R.J., Ambrose D. (2021). A new Model of Giftedness Emphasizing Active Concerned Citizenship and Ethical Leadership That Can Make a Positive, Meaningful, and Potentially Enduring Difference to the World. Conceptions of Giftedness and Talent.

[10] Ambrose D., Sternberg R.J., Ambrose D. (2021). Interdisciplinary Exploration Guiding Conceptions of Giftedness. Conceptions of Giftedness and Talent.

[11] Diamond A., McCardle P., Freund L., Griffin J.A. (2016). Why assessing and improving executive functions early in life is critical. Executive Function in Preschool-Age Children: Integrating Measurement, Neurodevelopment and Translational Research.

[12] Viana-Sáenz L., Sastre-Riba S., Urraca-Martínez M.L. (2021). Executive Function and Metacognition: Relations and Measure on High Intellectual Ability and Typical Children. Sustainability.

[13] Runco M.A., Ambrose D., Cross T. (2009). The Continuous Nature of Moral Creativity. Morality, Ethics and Gifted Minds.

[14] Sternberg R.J., Ambrose D. (2021). Conceptions of Giftedness and Talent.

[15] Gómez C., Gómez Y.C., Muguerza J. (2019). El ámbito de la moralidad: Ética y moral. La Aventura de la Moralidad (Paradigmas, Fronteras y Problemas de la Ética).

[16] Tirri K. (2010). Combining Excellence and Ethics: Implications for Moral Education for the Gifted. Roeper Rev..

[17] Sternberg R.J. (2009). Ethics and giftedness. High Abil. Stud..

[18] Mumford M.D., Higgs C.A. (2020). Leader Thinking Skills: Capacities for Contemporary Leadership.

[19] Tirri K., Sternberg R.J., Ambrose D. (2016). Holistic Perspectives on Gifted Education for the 21st Century. Giftedness and Talent in the 21st Century: Adapting to the Turbulence of Globalization.

[20] Lovecky D.V., Ambrose D., Cross T. (2009). Moral Sensitivity in Young Gifted Children. Morality, Ethics and Gifted Minds.

[21] Narváez D., Endicott L. (2009). Ethical Sensitivity, Nurturing Character in the Classroom.

[22] Rest J.R., Narvaez D., Thoma S.J., Bebeau M.J. (1999). DIT2: Devising and testing a revised instrument of moral judgment. J. Educ. Psychol..

[23] Tirri K., Nockelaien P. (2011). Measuring Multiple Intelligences and Moral Sensitivities in Education.

[24] Ambrose D., Cross T. (2009). Morality, Ethics and Gifted Minds.

[25] Schutte I., Wolfensberger M., Tirri K. (2014). The Relationship between Ethical Sensitivity, High Ability and Gender in Higher Education Students. Gift. Talent. Int..

[26] Ambrose D., Ambrose D., Cross T. (2009). Morality and High Ability: Navigating a Landscape of Altruism and Malevolence. Morality, Ethics and Gifted Minds.

[27] Calero M.D., García-Martín M.B., Jiménez M.I., Kazén M., Araque A. (2007). Self-regulation advantage for high-IQ children: Findings from a research study. Learn. Individ. Differ..

[28] Roeper A., Silverman L.K., Ambrose D., Cross T. (2009). Giftedness and Moral Promise. Morality, Ethics and Gifted Minds.

[29] Urraca-Martínez M.L., Sastre-Riba L., Viana-Pérez L. (2021). World Perception and High Intellectual Ability: A Comparative Study. Psicol. Educ..

[30] Sternberg R.J. (2012). Giftedness and ethics. Gift. Educ. Int..

[31] Cámara-Pastor T. (2021). Sensitividad Ética: Un Estudio Comparativo Entre Escolares con Alta Capacidad Intelectual y Escolares Típicos.

[32] Narvaez D., Getz I., Rest J.R., Thoma S.J. (1999). Individual moral judgment and cultural ideologies. Dev. Psychol..

[33] Crowson H.M. (2004). Human Rights Attitudes: Dimensionality and Psychological Correlates. Ethics Behav..

[34] Ramos F.J., Hernández-Mendo A., Pastrana J.L., Blanco-Villaseñor A. (2012). SAGT: Software para la Aplicación de la Teoría de la Generalizabilidad. Final Project on Technical Engineering in Computer Managing of the Higher Technical School of Computer Engineering.

[35] Sternberg R.J., Ambrose D. (2016). Renzulli, Stream of Consciousness on Creativity, Globalization, Technology, and What Is Happening in a Rapidly Changing World. Giftedness and Talent in the 21st Century: Adapting to the Turbulence of Globalization.

[36] Piechowski M.M., Ambrose D., Cross T. (2009). The inner world of the young and bright. Morality, Ethics and Gifted Minds.

